# *miRegulome*: a knowledge-base of miRNA regulomics and analysis

**DOI:** 10.1038/srep12832

**Published:** 2015-08-05

**Authors:** Debmalya Barh, Bhanu Kamapantula, Neha Jain, Joseph Nalluri, Antaripa Bhattacharya, Lucky Juneja, Neha Barve, Sandeep Tiwari, Anderson Miyoshi, Vasco Azevedo, Kenneth Blum, Anil Kumar, Artur Silva, Preetam Ghosh

**Affiliations:** 1 Centre for Genomics and Applied Gene Technology, Institute of Integrative Omics and Applied Biotechnology (IIOAB), Nonakuri, Purba Medinipur, WB-721172, India; 2 Department of Computer Science, Virginia Commonwealth University, Richmond, VA-23284, USA; 3 School of Biotechnology, Devi Ahilya University, Khandwa Road Campus, Indore, MP, India; 4 Laboratorio de Genetica Celular eMolecular, Departmento de Biologia Geral, Instituto de Ciencias Biologics, Universidade Federal de Minas Gerais CP 486, CEP 31270-901 Belo Horizonte, Minas Gerais, Brazil; 5 Department of Psychiatry and McKnight Brain Institute, University of Florida, College of Medicine, Gainesville, Florida, USA; 6 Instituto de Ciências Biológicas, Universidade Federal do Pará, Rua Augusto Corrêa, 01 - Guamá, Belém, PA, Brazil

## Abstract

miRNAs regulate post transcriptional gene expression by targeting multiple mRNAs and hence can modulate multiple signalling pathways, biological processes, and patho-physiologies. Therefore, understanding of miRNA regulatory networks is essential in order to modulate the functions of a miRNA. The focus of several existing databases is to provide information on specific aspects of miRNA regulation. However, an integrated resource on the miRNA regulome is currently not available to facilitate the exploration and understanding of miRNA regulomics. *miRegulome* attempts to bridge this gap. The current version of *miRegulome* v1.0 provides details on the entire regulatory modules of miRNAs altered in response to chemical treatments and transcription factors, based on validated data manually curated from published literature. Modules of *miRegulome* (upstream regulators, downstream targets, miRNA regulated pathways, functions, diseases, etc) are hyperlinked to an appropriate external resource and are displayed visually to provide a comprehensive understanding. Four analysis tools are incorporated to identify relationships among different modules based on user specified datasets. *miRegulome* and its tools are helpful in understanding the biology of miRNAs and will also facilitate the discovery of biomarkers and therapeutics. With added features in upcoming releases, *miRegulome* will be an essential resource to the scientific community. Availability: http://bnet.egr.vcu.edu/miRegulome.

microRNAs (miRNAs) are small non-coding RNAs that inhibit post-transcriptional gene expression by complementary base pairing at the 3′-UTRs of target messenger RNAs (mRNAs)^1^. Transcription of a miRNA coding gene is under direct control of transcription factors (TFs). Expression of a miRNA is also regulated by environmental factors, xenobiotics, and drugs. These factors essentially regulate TFs and consequently regulate transcription of miRNAs^2^. A TF can positively or negatively regulate miRNA transcription. A transcribed miRNA, by virtue of its feed-back and feed-forward loop regulation mechanisms, may regulate its own transcription machinery or the expression of other genes and thereby impacts gene expression significantly^3^. A single miRNA may target nearly 200 mRNAs^4^ and henceforth may regulate multiple signalling pathways and various essential biological processes (BPs) such as development^5^, aging^6^, immunity, and autoimmunity^7^, etc.

Deregulations of miRNAs are well documented for their association with various patho-physiological conditions including different types of cancers^8^, metabolic disorders^9^, and neuronal diseases^10^ among others. Therefore, understanding miRNA regulation is highly important in bio-medical research. Exploration of the entire regulome of a miRNA is indispensable in understanding its biology and mechanisms through which it regulates gene expression under a given biological condition. This will also help in the development of diagnostic, prognostic, and therapeutic strategies^11^,^12^,^13^. The regulome of a miRNA essentially consists of modules such as upstream regulators, downstream targets, miRNA modulated pathways, and regulated BPs. The regulome also considers associated diseases when a miRNA is deregulated. A schematic of our proposed miRNA regulome is presented in Fig. 1.

Several existing miRNA-related databases individually provide information on specific aspects of a miRNA. For example, miRbase^14^ maintains data on sequence repositories, mir2Disease^15^ provides miRNA-disease relationships, TransmiR^16^ maintains information on miRNAs and their upstream TFs, and miREnvironment^17^ offers information on miRNA regulation in response to environmental factors. Various databases such as miRecords^18^, miRWalk^19^, mirDIP^20^, miRTarBase^21^, etc, have been developed to enlist predicted and experimentally validated targets of miRNAs. However, none of these databases provide the entire regulome of a miRNA or are helpful in understanding the biology or function of miRNAs by analysing a stand-alone database. Attempts have been made to understand the miRNA interactome at a systems level in *C. elegans* (TF-miRNA-TF interactions)^22^ through computational simulation of miRNA regulated overall gene expression and cross-talk between miRNA targets^23^ and by constructing regulatory models of miRNA-kinase-TF, miRNA-TF, and TF-TF^24^. Similarly, systems approaches for predicting TF-miRNA crosstalk in human protein interactome^25^, demonstration of regulatory principles among miRNAs, TFs, and miRNA target genes^26^, miRNA-mRNA and miRNA-miRNA interactions^27^, and tissue-specific miRNA-TF regulatory networks^28^ have also been attempted to explore the miRNA interactome. However, these works are mostly computational predictions and do not provide the entire regulome of a miRNA.

The web-based resource, *miReg*^29^ provides basic correlations of various upstream regulators, downstream targets, BPs, and diseases of a miRNA based on experimentally validated data available in the PubMed literature. However, it is primitive in terms of data, completeness, functionality, and usability. Given the importance of miRNA in biomedical research, disease diagnosis, prognosis, and therapy, the huge inflow of new miRNA related information becomes a challenge. Therefore, a novel database that provides all the essential details of a miRNA regulome is necessary. Similarly, a state-of-the-art analysis platform to explore mechanisms behind various biological and patho-physiological processes that a miRNA regulates is also required.

*miRegulome* aims to address the need for such a novel database that represents the entirety of the miRNA regulome. In the current version of *miRegulome* (v1.0) we have incorporated all the downstream modules and TFs as well as the diverse group of chemicals as the upstream regulatory modules and their correlations. Several other aspects associated with the miRNA regulatory networks are also included (Fig. 1). The analysis tools for the current version of the miRNA regulome provide ranked association counts with Z-score statistical assessment for likely functions and disease associations of a set of input miRNAs. The list of resultant associated functions, diseases, and processes reported by our tools is similar to that provided by another tool that performs statistical enrichment analysis, TAM (http://210.73.221.6/tam).

## Construction and Contents

In *miRegulome* v1.0, we have incorporated experimentally validated data for all the downstream modules (targets, modulated pathways, regulated BPs, and associated diseases) and chemical and TFs as the upstream modules of a miRNA regulome. The data are manually curated from published literature indexed in PubMed. In the current version of *miRegulome*, physical, physiological, and mechanical upstream factors are not included. Also, this version contains most of the modules of miRNAs for human, mouse, and rat. But for other species’ miRNAs, mostly upstream modules (chemical or TF) are incorporated since the validated downstream modules of these miRNAs are not yet available in published literature. The step-by-step data collection process and their sources is represented in Supplementary Fig. S1 and the home page of *miRegulome* v1.0 is shown in Supplementary Fig. S2.

### Capturing miRNA and miRegulome Modules

#### miRNAs and upstream chemical regulators

In *miRegulome* v1.0, first we have focused on miRNAs that are either up- or down-regulated in response to a chemical (drugs, xenobiotics, carcinogens, organic and inorganic compounds, elements, metal, non-metals, and environmental factors etc.). PubMed literature database was extensively searched manually with key word combinations (for example: chemical + miR/miRNA/microRNA + regulation) to identify publications describing experimentally validated chemical-miRNA relationships. Each selected article having chemical-miRNA relationships was then manually curated to capture the (i) chemical(s) (ii) miRNAs responding to the chemical(s), (iii) species of the miRNAs, (iv) expression of the miRNAs (up-/down-regulation) in response to the chemical(s), (v) experimental conditions, (vi) techniques used to detect the expression levels of miRNAs, and (vii) the corresponding PubMed ID (Supplementary Fig. S3).

#### Upstream TF regulators and downstream targets

Apart from the upstream chemical regulators, TFs that regulate the transcription of a miRNA gene are one of the main classes of upstream regulators of the miRNA. Since miRNA’s basic function is to target specific mRNAs, such targets of miRNAs are the most important components in a miRNA regulome. In *miRegulome* v1.0, experimentally validated upstream TF regulators and the downstream target genes/mRNAs of each miRNA that have upstream chemical regulators, are manually curated from the PubMed indexed literature and incorporated into the database. Extensive manual search was carried out using specific key words such as target, regulator, transcription factor, “upregulates”, and “downregulates” along with the name of the miRNA having upstream chemical regulators (identified in the previous step/search) to capture these relationships (Supplementary Fig. S4A,B).

#### Prioritized targets and miRNA functions

The availability of prioritized targets and target-based top functionalities of a miRNA are unique features of *miRegulome*. We manually performed the target prioritization based on the number of interactions of a target in a protein-protein interaction network using the ToppNet algorithm of ToppGene suite^30^. Since miRNA regulates the expression of a gene, we used 11 house keeping genes as described by Eisenberg and Levanon^31^ in the training set and all experimentally validated targets of each miRNA (present in *miRegulome*) as the test set to perform the ToppNet analysis with its default parameters. Ranking or prioritized targets list were prepared and included in the *miRegulome* database based on the interaction counts of a target (higher count for higher rank). To assign the top functionalities of a miRNA, all the targets of a miRNA were subjected to ToppFun prediction analysis (with the default parameters) of the ToppGene suit^30^. In parallel, all the targets of that particular miRNA were analyzed using “Functional Annotation” module of DAVID^32^ with its default *p*-value cut off = 0.1. The first 25 common top ranked predicted functions (BPs) derived from both the tools are listed under “Function of miRNA” module of *miRegulome* (Supplementary Fig. S4C,D).

#### miRNA involved pathways

The second unique feature of *miRegulome* consists of linking a miRNA to the pathways, it regulates. In order to do so, all the validated targets of each miRNA were subjected to DAVID^32^ for enrichment into Kyoto Encyclopedia of Genes and Genomes (KEGG)^33^ pathways. The ten enriched pathways are listed under “Pathways” tab in the second table with corresponding KEGG links. In order to give a broader picture of the pathways in which the miRNA is involved, a “More” option is given at the end of the pathways list, each of which is hyperlinked to the corresponding miRNA pathways listed in the miRNAPath database^34^ (Supplementary Fig. S4E).

#### Disease module

The ultimate goal of miRNA research is to explore how a miRNA is associated with a patho-physiological process. Therefore, miRNA-disease association is an essential feature in a miRNA regulome. miRNA-disease relationships along with regulation of the miRNA (up- and down-regulation) in the disease condition were manually curated from PubMed listed published literature for those miRNAs that respond to chemical stimulus and were incorporated in *miRegulome* v1.0 under “Disease involvement” module. Similar to the “Pathways” module, a “More” option is given which is hyperlinked to that miRNA with all its associated diseases listed in the miR2Disease database^15^ (Supplementary Fig. S4F).

### Quality Control

To ensure the accuracy and consistency of the data before recording it to the *miRegulome* database, quality control (QC) checks were performed thrice. Three individual team members manually cross-checked the curated information such as validated TF regulators, chemicals, targets, conditions, techniques, diseases etc. and their associations with the miRNA using the particular PubMed publication. For predicted data (prioritized targets, miRNA top functions, pathways etc.), the cross-checks were carried out by three different team members using the same tools and their fixed parameters. Inconsistency in the data, if observed during QC checks was manually corrected and incorporated into the database.

### Database Contents

**Figure 2:**
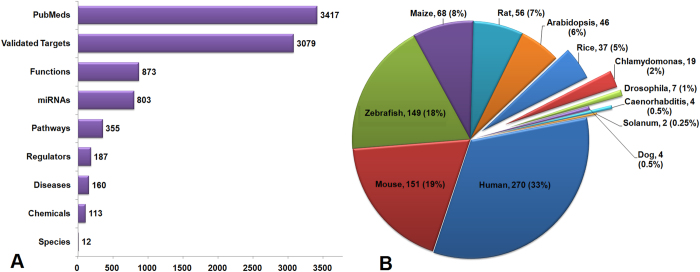
Distribution of *miRegulome* v1.0 contents. (**A**) Overall distribution of the database contents. (**B**) Species specific miRNA counts.

*miRegulome* v1.0 contains experimentally validated information for 803 miRNAs from 12 species, 113 chemicals, 187 upstream TF regulators, 3079 targets, and 160 diseases manually curated from 3417 PubMed indexed articles. Predicted 873 functions and 355 pathways are currently available in this database. The distribution of these data is represented in Fig. 2. We aim to update the database with manually curated new data every six months through partial automation.

### Database Design

*miRegulome* v1.0’s clean design helps users interact with the database in various ways. The regulome data has many-to-many relationships with its entities and this has been taken into consideration while designing the database. The database has been designed keeping the miRNA data as the central entity and all other database tables containing information about chemicals, functions, diseases, and genes are linked to the miRNA entry. Based on the current design, numerous specific combinations and associations among miRNAs, genes, conditions, techniques, diseases, and other related entities can be retrieved. This also allows retrieval of diverse nature of aggregated results based on dissimilar types of inputs the user may provide. The current design is beneficial in both - future approaches for analysis and incorporation of other tools and data into the existing system. The database design is presented in Supplementary Fig. S5.

### Visualization of miRNA Regulome

**Figure 3:**
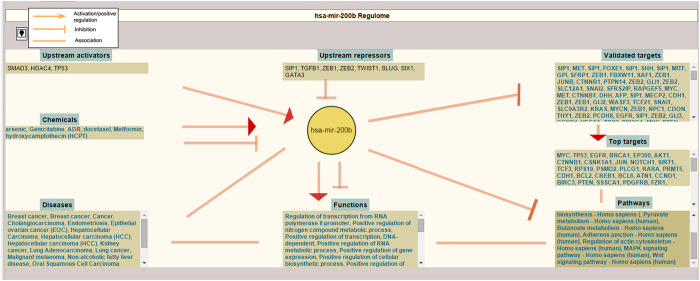
Visualization of miRNA regulome in *miRegulome* v1.0. The figure represents hsa-miR-200b regulome.

In order to represent the regulome of a miRNA, we developed an intuitive schematic visualization interface. Upon selection of a miRNA (under “miRNA Details” tab), the entire regulome of the selected miRNA will be visualized below the first table (Fig. 3). The schematic visualization provides the entire regulome of the miRNA with all its modules (chemicals, upstream activators and repressors, validated targets, enriched top targets, pathways, functions, and diseases) and their relationships with the miRNA. The relative impacts (activation, inhibition or association) of these modules on the miRNA are also graphically represented. JavaScript, HTML, and CSS are used to develop this complex interaction map in an intuitive way that is easy to interpret. This component is in addition to displaying the miRNA regulome information in a tabular format for better understanding of the miRNA regulome.

### Search Options and Description

Users can search the database in two different ways. The option of “Search by Chemical” can be used to retrieve miRNAs that are associated with a specific chemical. Upon selection of a chemical that is listed in alphabetical order in a dropdown menu, a table representing the miRNA-chemical relationships along with the species of the miRNAs, experimental conditions, effect of the chemical on the miRNAs, techniques used to detect the effects, and the corresponding PubMed ID etc. can be obtained. The user will be provided with the detailed regulome of the particular miRNA in a second table by clicking on any miRNA name under the last column (“miRNA Details” tab) of the first table. This table contains the detailed regulome where each tab is having a specific module and relationships related to the miRNA. The modules are “Upstream Regulators”, “Validated Targets”, “Prioritized/Top Targets”, “Function of miRNA”, “Pathways”, and “Disease Involvement”. To provide detailed information, each module is hyperlinked with suitable external resources. For instance, miRNAs are linked to miRBase^14^, chemicals to the Comparative Toxicogenomics Database (CTD)^35^, genes to Entrez (http://www.ncbi.nlm.nih.gov/gene), functions/BPs to EBI-GO (http://www.ebi.ac.uk/GOA), pathways to KEGG^33^ and miRNAPath^34^, and diseases are linked to miR2Disease database^15^ etc.

Using the “Advanced Search” option (Supplementary Fig. S6), the user can search information on all the twelve modules of the *miRegulome* v1.0 such as miRNA, regulators, targets, diseases etc. Importantly, the user can obtain specific relationships as per their requirements using a combination of query modules instantly (see the example under section “*Exploration of new biological events*”).

### Analysis Tools

*miRegulome* v1.0 is empowered with four unique tools to provide meaningful associations among chemical-disease, miRNA-disease, gene-disease, and disease-chemical-miRNA along with affected BPs based on user specific datasets. The results of these analyses correspond with a bipartite modelling approach which we developed to explore the associations among miRNAs and diseases available in the database. Maximum weighted matching algorithm is used to identify these associations^36^. In *miRegulome* v1.0, each association whether its chemical-miRNA, miRNA-disease, gene-miRNA etc. is manually curated from PubMed indexed literature and each of these relationships is tagged with specific PubMed ID from where the data are taken. These tools mine the database and give relevant associations to the user by querying the *miRegulome* database and counting the associations (direct or indirect) between entries. The output is returned as ranked association counts with Z-score statistical analysis rather than statistical enrichment measures. However, the results derived from these tools are quite similar to the ranked list of results returned by tools that use statistical enrichment and probability calculations (see the “Efficacy of *miRegulome* v1.0 tools” section). We calculated the Z-scores using the formula: *Z- score* = (*X* − μ)/σ, where ‘X’ is the association count of the particular association i.e. the number of PMIDs citing the association, ‘μ’ is the mean of the association counts for the entire association type, and ‘σ’ is the standard deviation. Hence, a positive Z-score indicates that the count of the association is higher than the average of such associations, and a negative value indicates that it is below the average. A value of 0 would mean it is equal to the average.

#### Chemical-disease analysis

This analysis tool allows the user to explore the associations between a chemical to a disease via miRNAs. When a user selects a particular chemical, the tool retrieves all the miRNAs associated with the chemical. Thereafter, the tool retrieves all the diseases in which the miRNAs are associated. For example, if a user selects chemical ‘*C2’* in the *Chemical-Disease* analysis tool, miRNAs *M1* and *M3* are retrieved and subsequently, their associated diseases *D1*, *D2* and *D3* are retrieved. Finally, the tool ranks the diseases in which these miRNAs (which are associated with the chemical) are associated, counting the PubMed IDs (Supplementary Fig. S7A,B). The tool then displays the disease names, their association counts and their respective Z-scores for the counts. Using similar methodology, the BPs which are associated with the miRNAs are displayed according to the count of their associations as recorded in the database. It does not assert a direct link between the chemical to a disease or to the BPs via the miRNA, rather allows the user to explore and test their hypothesis for indirect associations between the chemical and the disease via the miRNA.

#### miRNA-disease analysis

In this analysis, when an input of one or more miRNAs is provided, the tool provides three tables for the user to get a comprehensive understanding of their results. The tool searches for all diseases associated with the provided miRNA(s) and the distinct miRNA-disease associations (based on PubMed IDs) (Supplementary Fig. S7B). Following which, it ranks the diseases based on their number of recorded (PubMed IDs) associations and displays them in the Table A (Supplementary Fig. S8, Top affected diseases for given miRNAs). The user can click on the ‘*Count of PMIDs*’ and see the unique PubMed IDs supporting the results. The tool also displays the Z-scores for each disease along with its rank. Z-score is a standardized score for the count of each disease, indicating the resultant disease’s location in a distribution of other diseases, in relation to the mean and standard deviation of miRNA-disease counts. The Z-score of the disease tells the user, how many standard deviations it is from the mean of all miRNA-disease count distribution present in the database. To calculate this Z-score, we first calculated all the individual input miRNA and disease association counts and converted them to their respective Z-scores in Table C (Supplementary Fig. S8, Z-score for miRNA-disease associations). Thereafter, we added all the Z-scores associated to a single disease, thereby giving us the fair cumulative impact of all the input miRNAs with the single disease. This final cumulative Z-score is displayed for each disease. To further understand the relative impact of each miRNA (entered by the user) to the disease, we display Table C with each miRNA-disease edge with a Z-score. This value gives the user the individual miRNA-disease strength of association. It also displays which among the input miRNAs has the highest/lowest impact on the disease, thereby giving a more in-depth insight into the results displayed in Table A. Moreover, the ‘*Count of PMIDs*’ gives the cumulative count of PubMed IDs citing the associations of the input miRNAs with the disease. However, it does not take into account the impact of each miRNA-disease association towards the count. For e.g. a certain disease ‘*D*’ has ‘*Count of PMIDs*’ as 15 with miRNAs *M1, M2, and M3* associated with it. Among them, the the count of PubMed IDs for *M1-D1, M2-D1* and *M3-D1* are 12, 2, and 1 respectively. Evidently, here M1-D1 will have a higher positive Z-score because of its high count (assuming the *mean* is 5) and M3-D1 will have a negative Z-score. Nevertheless, when we add these individual Z-scores together, we get a fair relative scoring of the disease with respect to the input miRNAs, capturing the cumulative effect of the group of miRNAs. This is especially helpful, when the ‘*Count of PMIDs*’ for certain diseases are the same. In such cases, Z-score analysis would give a fine-grained ranking of the results. Table C explains the values obtained in Table A. This tool also searches the *miRegulome* database and identifies the most frequent BPs which are associated with the specified miRNA(s) and then displays them in Table B (Supplementary Fig. S8, Biological processes), ordered by rank, based on the count of associations present in this database.

#### Gene–disease analysis

When a list of genes is entered by the user in the input field, the tool searches for miRNAs associated with the set of genes and counts the number of gene-miRNA associations (i.e. PubMed IDs) recorded in the database. Thereafter, the tool searches and counts the existing relationships (i.e. PubMed IDs) between the observed miRNAs and diseases. Following which, the tool ranks the diseases based on their count of PubMed entries (Supplementary Fig. S9). Similarly, the tool also displays the list of BPs which are associated with the specified set of genes via miRNAs, and ranks them following the same principle of the miRNA-disease analysis tool. The tool does not assert a relationship between the entered genes and diseases but highlights the top diseases indirectly associated with the genes entered, via the miRNAs.

#### Disease-chemical/miRNA analysis

This tool works in the opposite way of the *Chemical – disease* analysis tool. It takes disease(s) as an input and searches the repository for specific miRNA(s)-disease associations. Thereafter, it retrieves the chemicals associated with the miRNAs. The tool displays these associations and ranks them based on the number of occurrences in the database (i.e. PubMed IDs). This gives the user an insight into possible role of chemicals in regulating miRNAs which are deregulated in the input disease(s).

## Utility and Discussion

### A single window for a wide range of data exploration

Information on validated upstream TF regulators can be obtained from TransmiR^16^, validated targets from miRWalk^19^, environmental factors acting on miRNAs from miREnvironment^17^, effects of small molecules on miRNA expression from SM2miR^37^, miRNA regulating pathways from miRNAPath^34^, and disease related miRNAs from miR2Disease^15^ databases. However, none of these databases provide additional information other than their respective specifics. Therefore, they are inadequate in terms of providing a comprehensive understanding of the miRNA regulome.

*miRegulome* is the first-of-its-kind integrated resource of miRNA regulomics having most of the modules of a miRNA regulome. Using *miRegulome* v1.0, in a single platform, the user can get almost all the information that is maintained by these databases along with several unique features such as prioritized targets and target based functional annotations of miRNAs among others. Therefore, it can be used in multiple ways to suit user needs.

Modules are hyperlinked to respective data resources so that if users are interested to explore additional information, it will ensure the comprehensive understanding of the data. Since, each miRNA regulating chemical is linked to the corresponding CTD webpage^35^; user can easily obtain the basic chemistry of the chemical and the details of gene interactions, associated diseases, other chemicals having comparable sets of interacting genes, BPs, pathways, etc. regulated by the chemical from the CTD (Supplementary Fig. S3). CTD, so far does not contain miRNA information for any chemical listed in the database. Therefore, the miRNA information of *miRegulome* for a chemical will be complementary to CTD thereby adding the entire range of regulatory network of the chemical. Similarly, basic information of a miRNA can be obtained by clicking on the name of the miRNA that is hyperlinked to miRBase^14^ and miRBase contains several useful information and links for the miRNA including nomenclature, basic annotation, stem-loop and mature sequences, locus report, Entrez, and HUGO Gene Nomenclature Committee (HGNC) (www.genenames.org) etc. From Entrez and HGNC, the user can get most of the resources which include associated published literature and even the clinically significant information on the miRNA. Since the targets and upstream TF regulators are hyperlinked to Entrez, user will also be able to get detailed information on these modules. Similarly, functions of miRNAs are hyperlinked to EBI-GO (http://www.ebi.ac.uk/GOA), pathways to KEGG^33^ and miRNAPath^35^, disease to miR2Disease database^15^ etc. (Supplementary Fig. S4). Further, additional miRNA resources and tools have also been listed under the “Resources” page of *miRegulome* (Supplementary Fig. S2). Therefore, using *miRegulome*, users can explore most of the information and analysis related to a miRNA.

### Visualization of regulome and data interpretation

**Figure 4:**
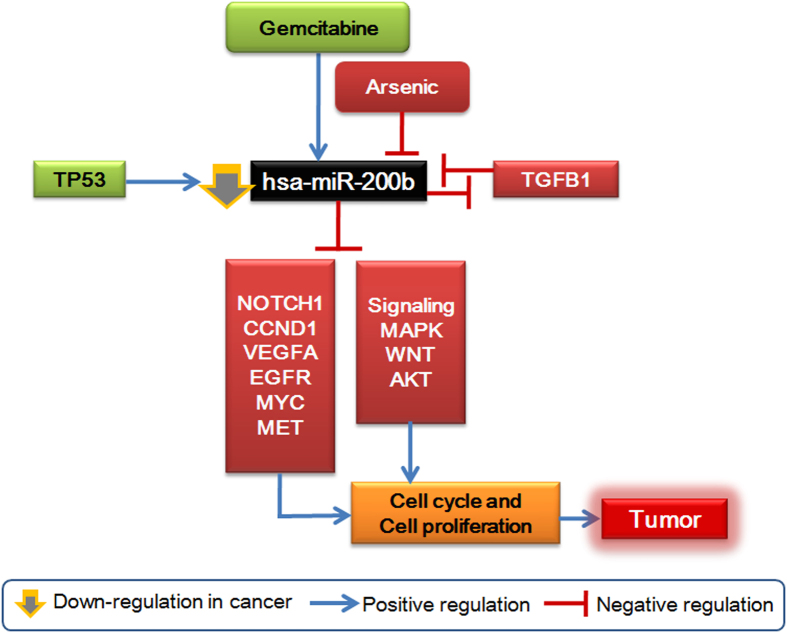
The relationships among various modules of hsa-mir-200b suggest that this miRNA is a probable tumor suppressor.

To simplify the understanding of a miRNA regulome, *miRegulome* v1.0 is integrated with an intuitive and effective schematic visualization tool. The complex interactions and relationships of a miRNA with its various modules can be visualized, thereby providing a cursory overview of the miRNA biology. This visual schematic is displayed when the user clicks on a miRNA under “miRNA Details” available in the first table. Fig. 3 represents visualization of hsa-miR-200b. The visualization gives a glimpse of seven modules (chemicals, upstream activators and repressors, validated targets, prioritized targets, biological pathways, BPs/functions, and disease associations) of the miRNA and the manner in which they regulate the miRNA or are being regulated by the miRNA with the relative impacts such as activation or inhibition or association. Further analysis of the visualization may provide deeper understanding of the mechanism and the impact of each module component on the hsa-miR-200b, and hence the biology and patho-physiological significance of the miRNA. Considering an example of cancer, from Fig. 3 and tabular forms of the regulome description, it can be observed that hsa-miR-200b: (i) forms a feed-back loop with TGFB1, (ii) P53 activates and TGFB1 inhibits its expression, (iii) inhibits MAPK, WNT, and AKT signalling pathways, (iv) inhibits cell cycle and cell proliferation by targeting cell cycle regulators and oncogenes like CCND1, VEGFA, MYC, NOTCH1, MET, EGFR etc. (v) is involved in cancer associated pathways, (vi) is downregulated in response to arsenic carcinogen, (vii) is upregulated by chemotherapy drug Gemcitabine and downregulated in Docetaxel-resistant cancers, and (viii) is downregulated in several cancers (Fig. 4). Therefore, it may be implicated that hsa-miR-200b could be a tumor suppressor miRNA and may be a potential therapeutic for a wide range of cancers.

### Exploration of new biological events

Since *miRegulome* gives most of the information associated with a miRNA, the user can explore the molecular mechanism and precise role of a miRNA behind a normal BP or a patho-physiological process. Identification of the particular role of a miRNA may consecutively help in developing miRNA-based diagnostics and therapeutic strategies.

User can search *miRegulome* v1.0 based on chemical using simple “Search by chemical” option or can use the “Advanced search” to get specific information as per the input search combinations. Using the “Search by Chemical”, user can get the validated regulations of a list of miRNAs in response to a user defined chemical. Chemical responses to miRNAs for twelve species (Human, Mouse, Rat, Dog, Zebra fish, *Drosophila*, *C. elegans*, *Arabidopsis*, Maize, Rice, *Solanum*, and Chlamydomonas) can be explored using this feature. From the retrieved list of miRNAs, user can get the regulome having upstream regulators (chemicals and TFs), regulated BPs, pathways, and disease involvement of any selected miRNA (currently available for Human, Mouse, Rat) and therefore can analyze any event related to that miRNA. Current version of *miRegulome* does not provide other upstream regulators except chemicals for other species.

In the “Advanced search” option, each module of miRNA regulome along with species, regulations, technique and condition, totalling twelve options have been accommodated. Therefore, single or a combination of input keywords can be used to get highly selective information. For example, for a particular disease, we can easily get a list of all up- and down-regulated miRNAs separately, using a combination of two input fields: disease and regulation.

**Figure 5:**
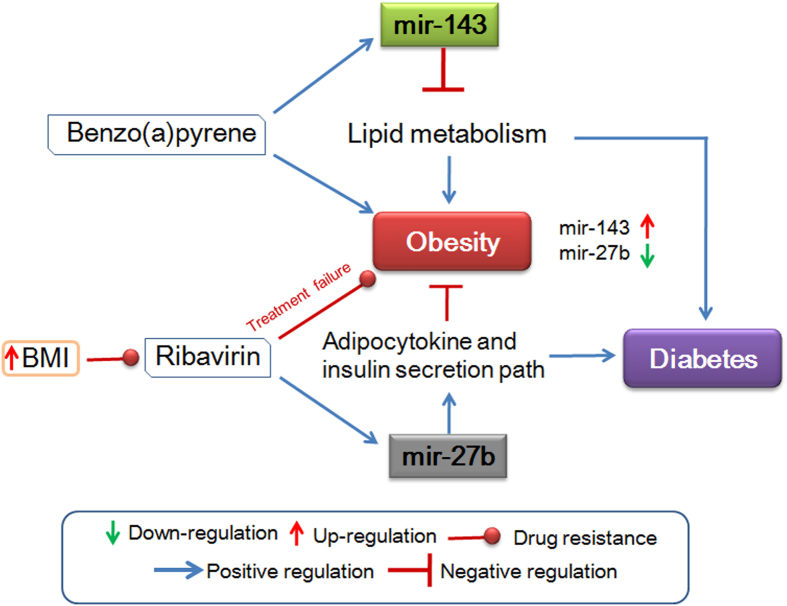
Emerging molecular mechanism and correlation between obesity and diabetes as identified by miRegulome data. For details, please see the text.

Similarly, we can explore complex associations among several modules and novel correlations using the “Advanced search” option. For example, it can be found that, hsa-mir-27b is down-regulated and hsa-mir-143 is up-regulated in obesity. Further, from *miRegulome* v1.0, it can also be established that hsa-mir-27b is involved in adipocytokine, insulin, and type-2 diabetes pathways and hsa-mir-143 acts in lipid metabolism pathway. These pathways are important events in obesity and therefore, deregulation of hsa-mir-27b and hsa-mir-143 may affect these pathways and eventually may lead to obesity and diabetes. Further, the database also provides correlation of obesity - mir-27b - Ribavirin and obesity - mir-143 - Benzo[a]pyrene. As per *miRegulome* v1.0, Benzo(a)pyrene and Ribavirin up-regulate mmu-mir-143 and hsa-mir-27b, respectively. It is reported that higher Body Mass Index (BMI) lowers bioavailability of Ribavirin and causes treatment failure in obese HCV patients^38^. On the other hand, Benzo[a]pyrene can induce obesity^39^. In summary, it can therefore be implicated that, (a) Benzo[a]pyrene upregulates mir-143 and affects lipid metabolism to induce obesity and (b) an aberrant expression of mir-27b may play a role in obesity-associated insulin resistance by modulating adipocytokines and Ribavirin resistance in obese patients. Similarly, it also suggests that these two miRNAs interlink obesity with diabetes at a new and deeper molecular level (Fig. 5). Therefore, *miRegulome* v1.0 may play an important role in exploring novel molecular mechanisms behind a disease as well as designing personalized medicine.

### miRegulome v1.0 tools and analysis

The user-friendly tools of *miRegulome* v1.0 can help the users to understand and test their hypotheses by exploring relationships among miRNA - function - disease, gene - function - disease, chemical - function - disease, and disease associated chemicals and miRNAs.

A user chosen set of miRNAs or genes can be used to understand relevant diseases and BPs associated with the sets using *miRNA-Disease* and *Gene-Disease* tool, respectively. Similarly, single disease or a set of diseases can be used as input to find the common miRNAs, chemicals, and BPs associated with the set or individual disease using *Disease-Chemical/miRNA* tool. In *miRNA-Disease* tool, users can search for species-specific (Human, Mouse, Rat) miRNA analysis using the corresponding prefix (has-, mmu-, rno-) for the miRNA symbols, or can adopt a combined analysis including all three species. For the latter, the user should not use species-specific prefix, instead should use general miRNA symbols such as miR-21 etc. *Disease-Chemical/miRNA* tool can provide the associated miRNAs, chemicals, and affected BPs for a single or a combination of diseases.

### Efficacy of miRegulome tools

To demonstrate the efficacy of *miRegulome* v1.0 tools, we used sets of miRNAs or genes that have been reported to show directly up- or down- regulated in a particular disease condition, and have been considered as biomarkers or signatures for the corresponding diseases. Ten such published datasets from ten PubMed literature (that are not incorporated so far in this version of *miRegulome*) were randomly selected and used for the analysis. The set of miRNAs or genes from each publication is fed into the corresponding tool and checked for the output results. Upon analysis, we look for the results if they match with the disease(s) mentioned in the corresponding publication for that particular set of miRNAs or genes. *miRegulome* v1.0 tools rank top 15 diseases associated with single or a set of miRNAs or gene(s) along with a count of association/count of PMIDs along with respective Z-scores based on the strategy as described in the “Analysis Tools” section. Out of the tested ten miRNA and 10 gene sets from twenty different publications, the *miRNA-Disease* and *Gene-Disease* association analysis tools are able to rank the diseases associated with the miRNA and gene sets as per the published literature within the top 15 diseases and mostly under the first five listed diseases.

Out of ten tested miRNA sets from ten different PubMed literature, in 7 cases the tool ranks the same disease associated with the miRNA set (as mentioned in the corresponding literature) within rank 5 and only 3 are between ranks 10 to 14 (Supplementary Table S1). As per the PubMed: 22213236^40^, a set of human miRNAs comprising of miR-21, miR-31, mir-122, miR-221, miR-222, miR-145, miR-146a, miR-200c, and miR-223 are deregulated in hepatocellular carcinomas. When this set of miRNA is used for analysis, the *miRNA-Disease* analysis tool gives hepatocellular carcinoma at rank-3 with an association score/count of PMIDs 50 (Z-score: 37.821). Another set of miRNA miR-1, miR-134, miR-186, miR-208, miR-223, and miR-499 are associated with acute myocardial infarction according to PubMed: 23641832^41^. When this set is used in *miRNA-Disease* analysis tool, the outcome ranks myocardial infarction at position 2 with an association score/count of PMIDs 6 (Z-score: 4.222). Additionally, the associated BPs ranked by the tool also correlate with the diseases; supporting the efficacy of the tool in associating diseases with the input miRNA sets (Supplementary Fig. S8).

The performance of miRegulome tools may be compared to tools that perform statistical enrichment analysis. We compared the results from *miRNA-Disease* analysis tool with the **T**ool for **A**nnotations of human **m**iRNAs (TAM) (http://210.73.221.6/tam)^42^ that calculates the probability of a particular miRNA belonging to a cluster of miRNAs, using its expression values and gives enriched miRNA-associated disease and functions based on *p*-values. *miRegulome* v1.0 does not have expression values or information of cluster of miRNA families and thereby cannot do statistical significance analysis. However, we have used the Z-score statistical assessment of the results which gives a standardized scoring metric and credibility to the miRNA-disease associations. For comparison, we used the same miRNA sets (Supplementary Table S1), that are used to test efficacy of *miRNA-Disease* analysis tool and selected overrepresentation and set version 2 of TAM. As shown in Supplementary Table S2, the ranking of diseases for a particular set of miRNA based on *p*-values by TAM is comparable to the ranking by our *miRNA-Disease* analysis tool; although the absolute ranks differ. This variation could be due to more disease entries used by TAM analysis (as it uses the entire HMDD database^43^).

Supplementary Table S3 shows the results for the *Gene-Disease* association analysis tool. It is observed that, a 29-gene signature for lung cancer (PubMed: 19951989)^44^ gives the same cancer at rank-5 with an association score 215. Two genes COL2A1 and ATP4B identified as markers in gastric cancer (PubMed: 23606240)^45^ gives colorectal cancer as the rank-1 disease with an association score of 99. For the *Gene-Disease* tool, we observed that, in 50% cases the tool ranks the disease associated with the gene set within rank 5 and 100% within rank 10 (Supplementary Table S3). Similar to the *miRNA-Disease* analysis, the ranked BPs also correlate with the disease.

We have also tested the *Chemical-Disease* and *Disease-Chemical/miRNA* association tools and have received similar precision in results (data not shown). Therefore, these tools are useful in predicting or identifying disease associations or disease associated miRNAs/genes/chemicals for user specified data sets e.g., (i) miRNA/gene biomarkers for a disease, (ii) disease susceptibility in response to a chemical based on miRNA profile, (iii) miRNA/gene signature of a disease, (iv) affected or regulated BPs by a group of miRNA/gene, and (v) developing therapeutic strategies.

## Conclusions and Future development

*miRegulome* may be considered as the advanced version of *miReg*^29^ in terms of data, functionality, usability, and completeness. *miRegulome* aims to provide the complete regulome of any miRNA listed in this database as derived from published literature. The current version of *miRegulome* v1.0 provides the complete regulome for chemically responsive 803 miRNAs from 12 species. However, the miRNAs from Human, Mouse, and Rat currently have most of the downstream modules of their regulome. For other species, as most of these modules are unavailable in published literature, they are partially available here. Once the data for these missing modules are available in the public domain, they will be incorporated in the upcoming versions of *miRegulome*.

Performance of *miRegulome* v1.0 tools depend on the available data present in the database. Since these tools work based on the association counts; the efficacy of their analysis can be further improved if more data are added to the database. For the next release, we aim to add new data and new upstream regulators (such as physical, physiological and mechanical factors) for the existing miRNAs. Similarly, miRNAs from new species will also be included to further enrich the database. New modules such as regulatory relationships between miRNAs and epigenetic modifications, miRNAs and disease related SNPs in their target genes, and variations in miRNA sequence and its associated phenotypes among others will be included. Interactions among diseases can be explored using miRNA-disease relation model of the current data. This can be achieved by representing the miRNA-disease information as a network. Further work would also aim to develop novel ways of interaction with the database and ascertain the nature of associations among different diseases, in which the effect of a certain disease on the occurrence or receding of other diseases can be evaluated. Tools for prediction of prognostic and early diagnostic markers will also be developed. In order to achieve that, high throughput genome-wide expression and association data will be incorporated in the next versions of *miRegulome* and the novel “reverse transcriptomics” approach by Barh *et al.*^46^ will be implemented. Similarly, disease specific therapeutic miRNAs and small molecules targeting the disease causing precursor microRNAs also will be included. Further, the miRNAs may be classified based on -3p and -5p and the new version will be developed based on such strand specific miRNA information. We also aim to develop a better visualization and advanced integrated analysis system for next-level of interaction of *miRegulome* modules and interpretation of miRNA functions in various biological and patho-physiological processes and to understand if miRNA-miRNA direct interactions do also exist.

## Availability and requirements

*miRegulome* v1.0 can be accessed online at http://bnet.egr.vcu.edu/miRegulome and is free for academic research. Commercial use is not permitted. jQuery, JavaScript, and HTML have been used to design the user interface. PHP has been used for server-side scripting support. Google visualization library has been used to represent the data in an interactive form. MySQL v.5.1.63 is used as database to store regulome information. It runs on Apache 2.2.17 on Ubuntu 12.04. The database and its integrated tools can be best used using the latest versions of Google Chrome and Mozilla Firefox browsers.

## Additional Information

**How to cite this article**: Barh, D. *et al.* *miRegulome*: a knowledge-base of miRNA regulomics and analysis. *Sci. Rep.* **5**, 12832; doi: 10.1038/srep12832 (2015).

## Supplementary Material

Supplementary Information

## Figures and Tables

**Figure 1:**
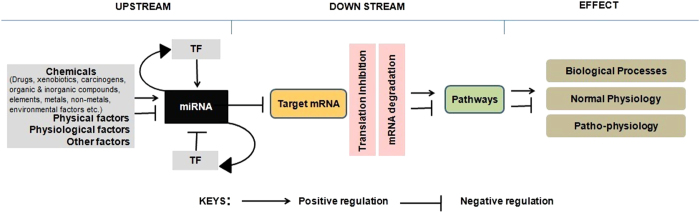
Schematic diagram of modules and their inter-relationships in a miRNA regulome.
