# Supplementary material for: miRegulome: a knowledge-base of miRNA regulomics and analysis
Source: Sci Rep. 2015 Aug 5;5:12832. doi: 10.1038/srep12832 (PMC4525332; doi:10.1038/srep12832)
Supplement: Supplementary Information [file srep12832-s1.pdf]

Supplementary information

miRegulome: a knowledge-base of miRNA regulomics and analysis

Debmalya Barh[1]*, Bhanu Kamapantula[2†], Neha Jain[1,3±], Joseph Nalluri[2†], Antaripa Bhattacharya[1], Lucky Juneja[1,3±], Neha Barve[1,3], Sandeep Tiwari[1,4], Anderson Miyoshi[4], Vasco Azevedo[4], Kenneth Blum[1,5], Anil Kumar[3], Artur Silva[6], Preetam Ghosh[2]

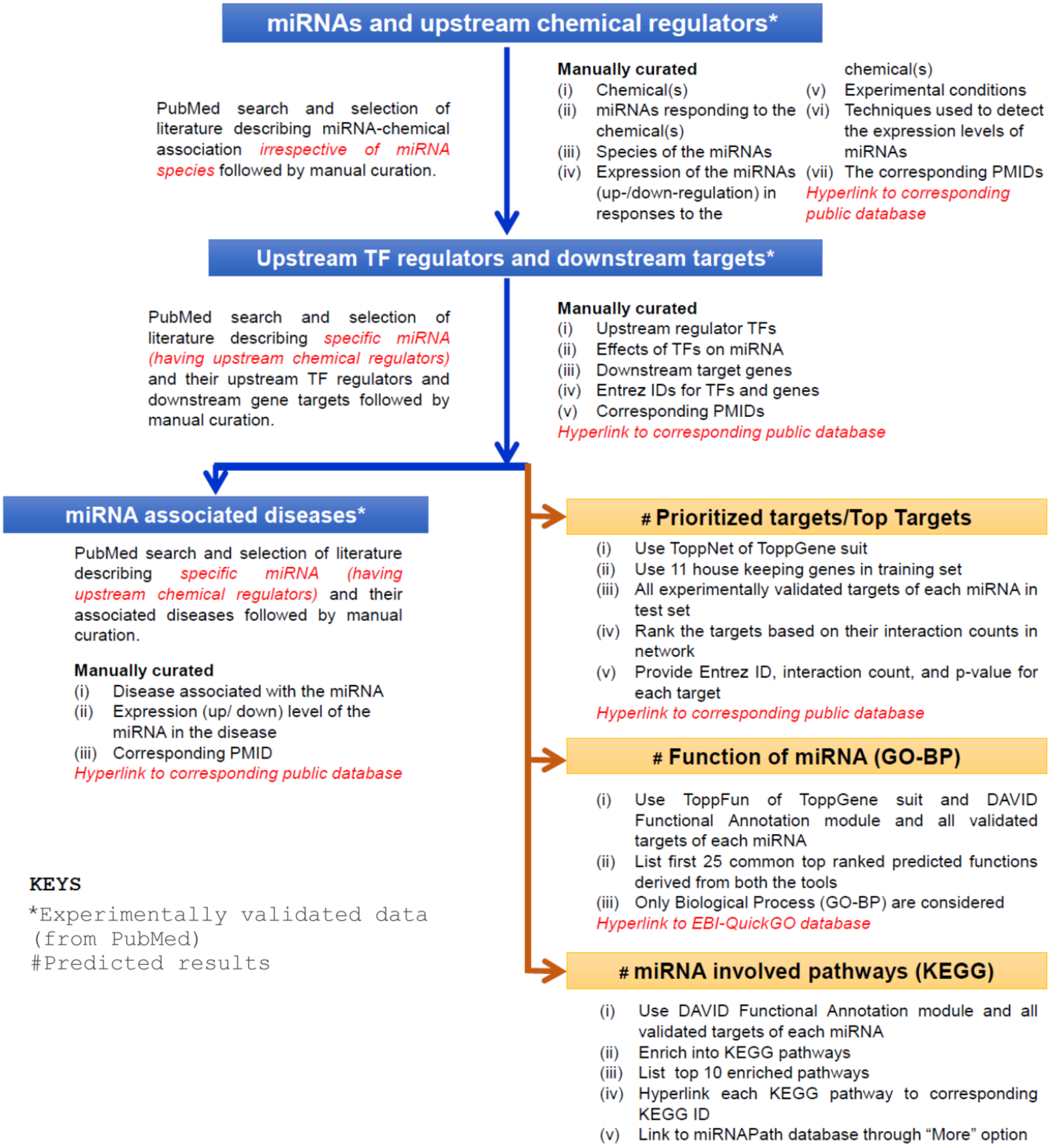

Supplementary Figure S1: The step-by-step data collection process and their sources to develop *miRegulome* v1.0.

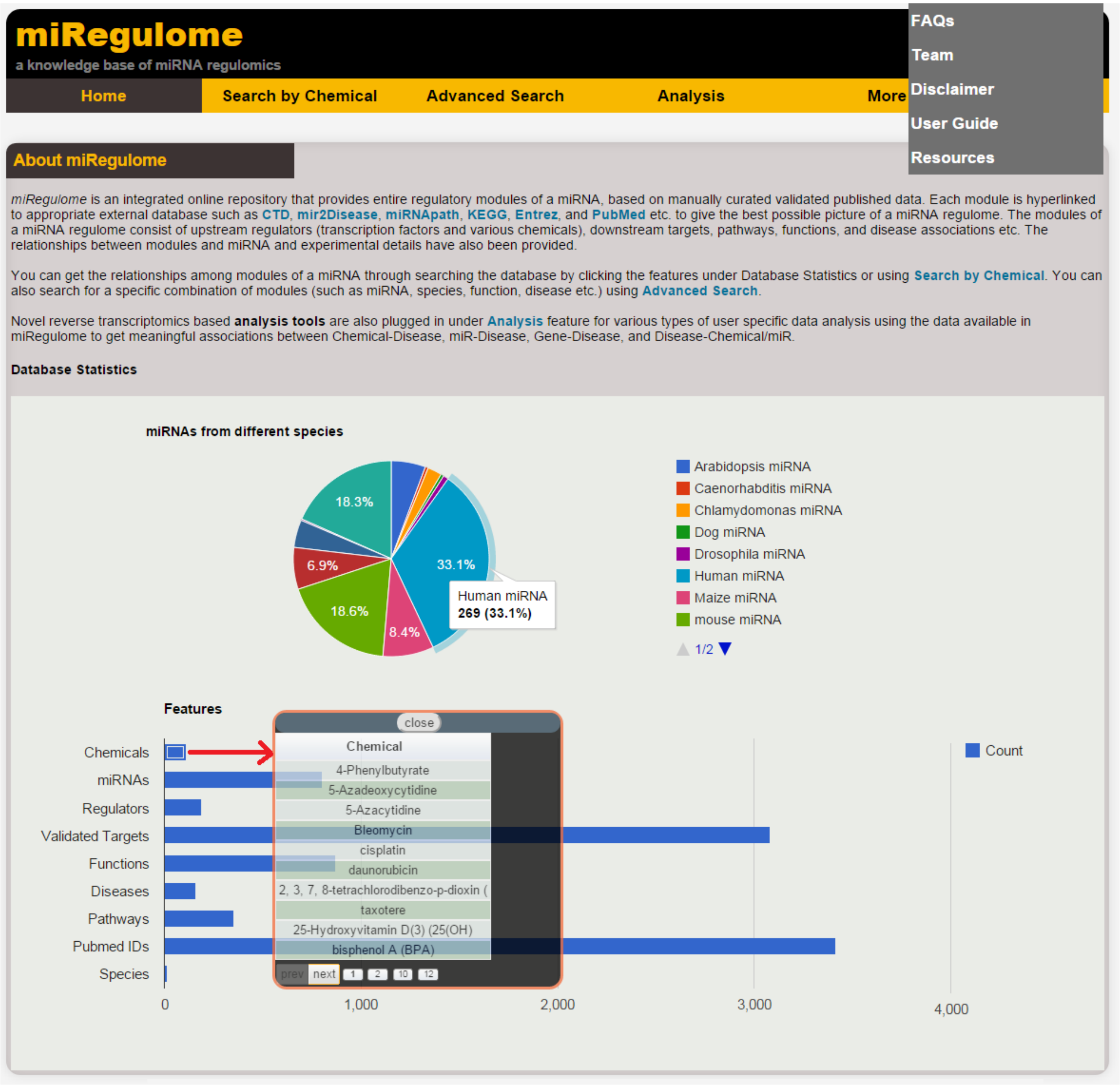

Supplementary Figure S2: The home page of *miRegulome* v1.0. The page provides the database content statistics in graphical format and also the direct links to chemical-based search.

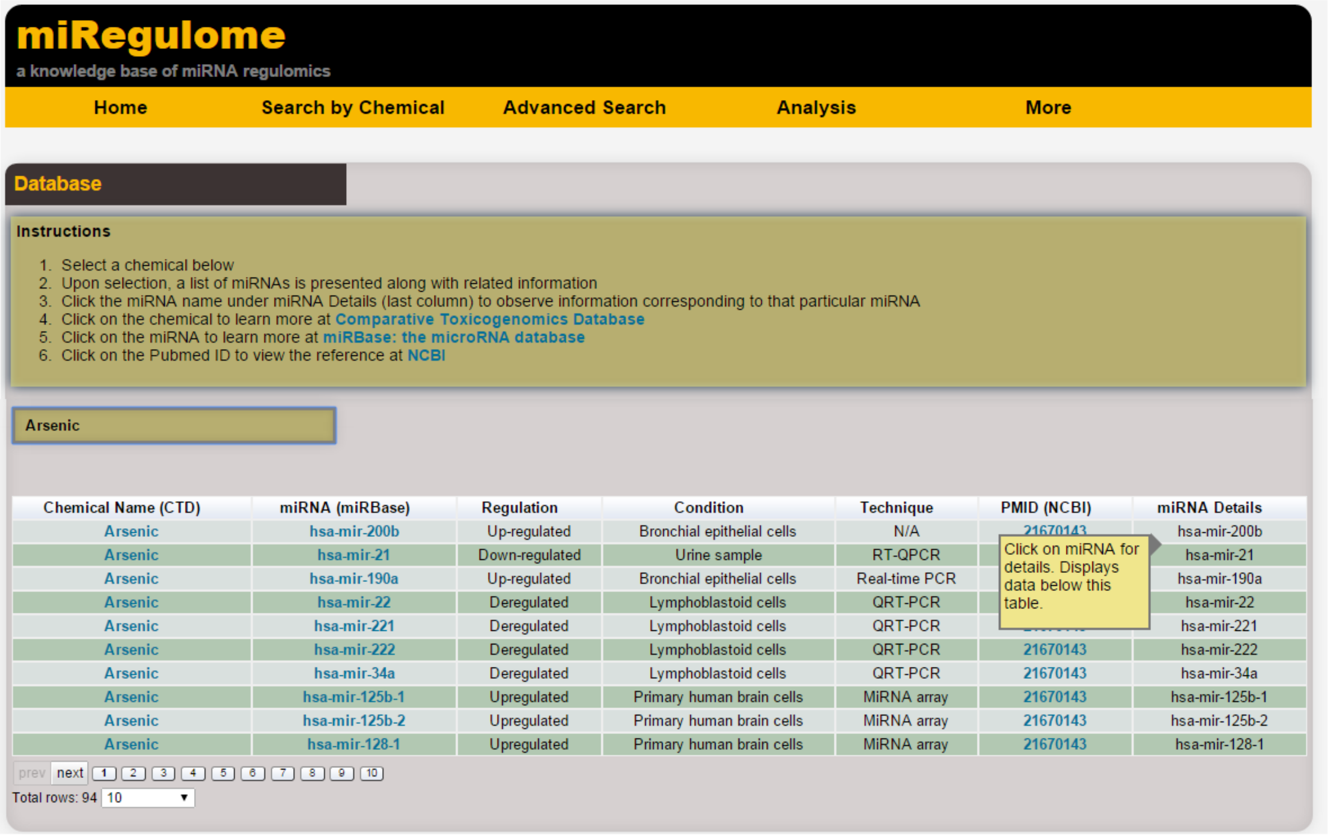

miRegulome
a knowledge base of miRNA regulomics

Home | Search by Chemical | Advanced Search | Analysis | More

Database

Instructions

1. Select a chemical below
2. Upon selection, a list of miRNAs is presented along with related information
3. Click the miRNA name under miRNA Details (last column) to observe information corresponding to that particular miRNA
4. Click on the chemical to learn more at Comparative Toxicogenomics Database
5. Click on the miRNA to learn more at miRBase: the microRNA database
6. Click on the Pubmed ID to view the reference at NCBI

Arsenic

Chemical Name (CTD)	miRNA (miRBase)	Regulation	Condition	Technique	PMID (NCBI)	miRNA Details
Arsenic	hsa-mir-200b	Up-regulated	Bronchial epithelial cells	N/A	21670143	hsa-mir-200b
Arsenic	hsa-mir-21	Down-regulated	Urine sample	RT-QPCR	[illegible]	hsa-mir-21
Arsenic	hsa-mir-190a	Up-regulated	Bronchial epithelial cells	Real-time PCR	[illegible]	hsa-mir-190a
Arsenic	hsa-mir-22	Deregulated	Lymphoblastoid cells	QRT-PCR	[illegible]	hsa-mir-22
Arsenic	hsa-mir-221	Deregulated	Lymphoblastoid cells	QRT-PCR	[illegible]	hsa-mir-221
Arsenic	hsa-mir-222	Deregulated	Lymphoblastoid cells	QRT-PCR	21670143	hsa-mir-222
Arsenic	hsa-mir-34a	Deregulated	Lymphoblastoid cells	QRT-PCR	21670143	hsa-mir-34a
Arsenic	hsa-mir-125b-1	Upregulated	Primary human brain cells	MiRNA array	21670143	hsa-mir-125b-1
Arsenic	hsa-mir-125b-2	Upregulated	Primary human brain cells	MiRNA array	21670143	hsa-mir-125b-2
Arsenic	hsa-mir-128-1	Upregulated	Primary human brain cells	MiRNA array	21670143	hsa-mir-128-1

Click on miRNA for details. Displays data below this table.

prev next 1 2 3 4 5 6 7 8 9 10
Total rows: 94 10

Supplementary Figure S3: Tabular form of chemical-miRNA relationships. Manual curation of (i) chemical(s) (ii) miRNAs responding to the chemical(s), (iii) species of the miRNAs, (iv) expression of the miRNAs (up-/down-regulation) in response to the chemical(s), (v) experimental conditions, and (vi) techniques used to detect the expression levels of miRNAs. The corresponding PubMed ID from which the experimentally validated data are curated is also mentioned. The chemical is hyperlinked to Comparative Toxicogenomics Database (CTD), miRNAs hyperlinked to miRBase, and PMID is hyperlinked to corresponding NCBI PubMed.

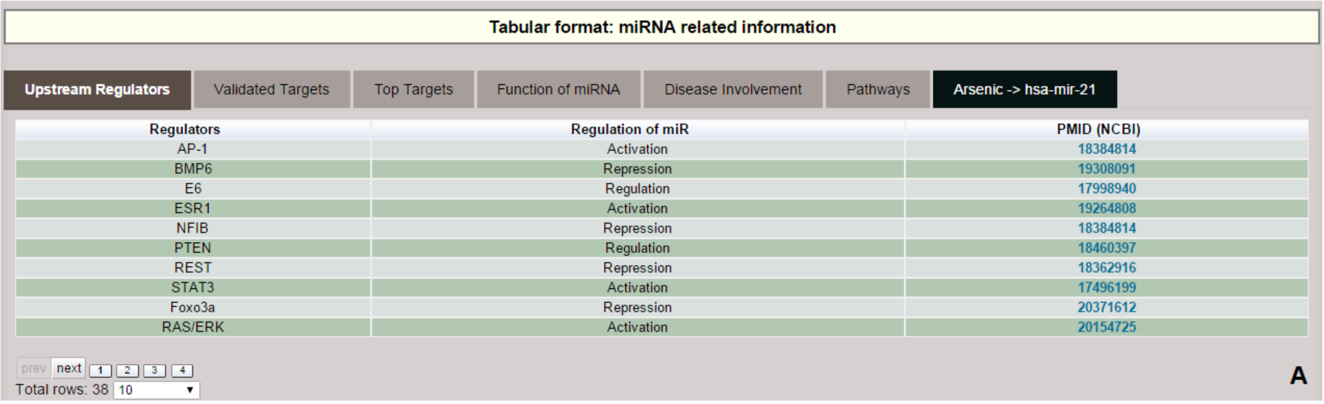

Tabular format: miRNA related information

Upstream Regulators | Validated Targets | Top Targets | Function of miRNA | Disease Involvement | Pathways | Arsenic -> hsa-mir-21

Regulators	Regulation of miR	PMID (NCBI)
AP-1	Activation	18384814
BMP6	Repression	19308091
E6	Regulation	17998940
ESR1	Activation	19264808
NFIB	Repression	18384814
PTEN	Regulation	18460397
REST	Repression	18362916
STAT3	Activation	17496199
Foxo3a	Repression	20371612
RAS/ERK	Activation	20154725

prev next 1 2 3 4
Total rows: 38 10

A

Tabular format: miRNA related information

Upstream Regulators | **Validated Targets** | Top Targets | Function of miRNA | Disease Involvement | Pathways | Arsenic -> hsa-mir-21

Gene Name (NCBI)	Entrez ID (NCBI)	PMID (NCBI)
ROS1	6098	21350856
DICER1	23405	18723136
PAK3	5063	17991735
PTEN	5728	19641183
PAH	5053	20110569
CD2	914	19831385
EGFR	1956	19493678
THY1	7070	21176238
ROS1	6098	19158092
LITAF	9516	20956972

prev next 1 2 10 60 69
Total rows: 687 10

B

Tabular format: miRNA related information

Upstream Regulators | Validated Targets | **Top Targets** | Function of miRNA | Disease Involvement | Pathways | Arsenic -> hsa-mir-21

Gene Rank	Gene Name (NCBI)	Entrez ID (NCBI)	Interaction	Score
1	ESR1	2099	830	2.59E-03
2	MYC	4609	580	1.80E-03
3	TP53	7157	569	1.38E-03
4	EGFR	1956	445	1.23E-03
5	BRCA1	672	399	8.87E-04
6	NPM1	4869	363	8.85E-04
7	SMAD3	4088	343	6.77E-04
8	SOX2	6657	328	6.21E-04
9	ILF3	3609	291	7.30E-04
10	SMAD2	4087	268	5.97E-04

prev next 1 2 10 30 33
Total rows: 324 10

C

Tabular format: miRNA related information

Upstream Regulators | Validated Targets | Top Targets | **Function of miRNA** | Disease Involvement | Pathways | Arsenic -> hsa-mir-21

Function of miRNA (Quick GO)
Positive regulation of macromolecule metabolic process
Positive regulation of RNA metabolic process
Regulation of programmed cell death
Regulation of cell death
Regulation of apoptosis
Positive regulation of transcription, DNA-dependent
Positive regulation of gene expression
Regulation of cell proliferation
Positive regulation of cellular biosynthetic process
Positive regulation of macromolecule biosynthetic process

prev next 1 2 3 4 5
Total rows: 45 10

D

Tabular format: miRNA related information

Upstream Regulators | Validated Targets | Top Targets | Function of miRNA | **Disease Involvement** | Pathways | Arsenic -> hsa-mir-21

Disease Name (mir2disease)	Regulation of miRNA	PMID (NCBI)
Acute lymphoblastic leukemia (ALL)	Down-regulated	18056805
Acute myeloid leukemia (AML)	Up-regulated	18056805
Autism spectrum disorder (ASD)	Up-regulated	18563458
Bladder cancer	Up-regulated	19487295
Bladder cancer	Up-regulated	19843843
Breast cancer	Up-regulated	16461460
Breast cancer	Up-regulated	18270520
Breast cancer	Up-regulated	18812439
Breast cancer	Up-regulated	18932017
Breast cancer	Up-regulated	17072344

prev next 1 2 10 14
Total rows: 136 10

E

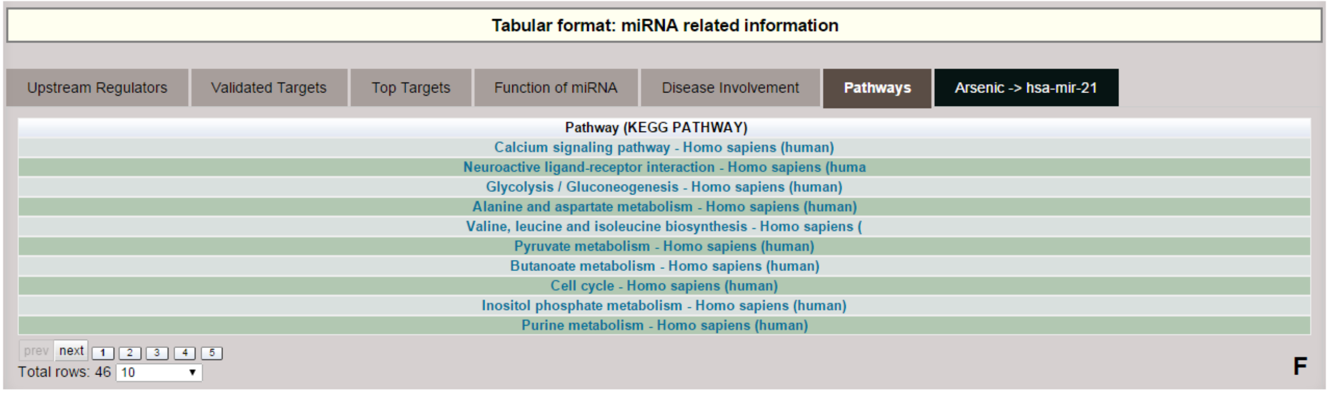

Supplementary Figure S4: Tabular form of miRNA hsa-mir-21 and its relationships with **(A)** upstream TF regulators, **(B)** validated targets, **(C)** top/prioritized targets, function (BP) of miRNA, **(E)** disease involvement, and **(F)** pathways in which the miRNA is involved. Clicking on a miRNA in the last column i.e. "*miRNA Details*" of the **Supplementary Figure S3** will give this information along with PMIDs and appropriate external database links to corresponding module.

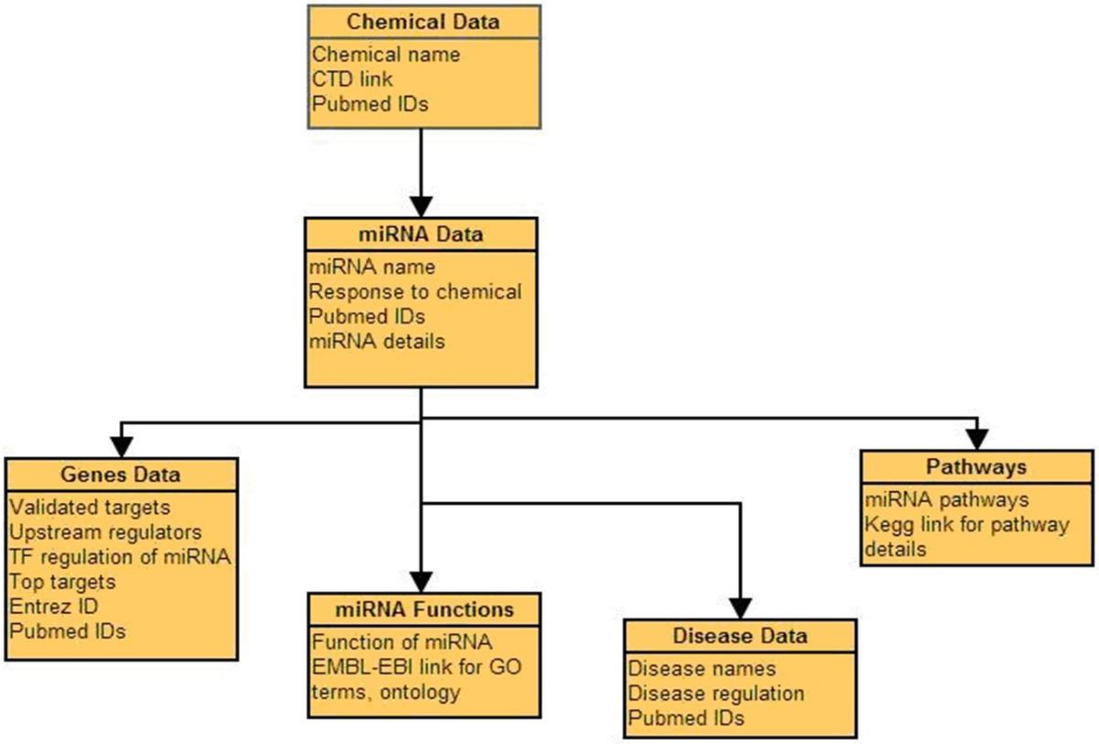

Supplementary Figure S5: The design/schema of *miRegulome* v1.0 database

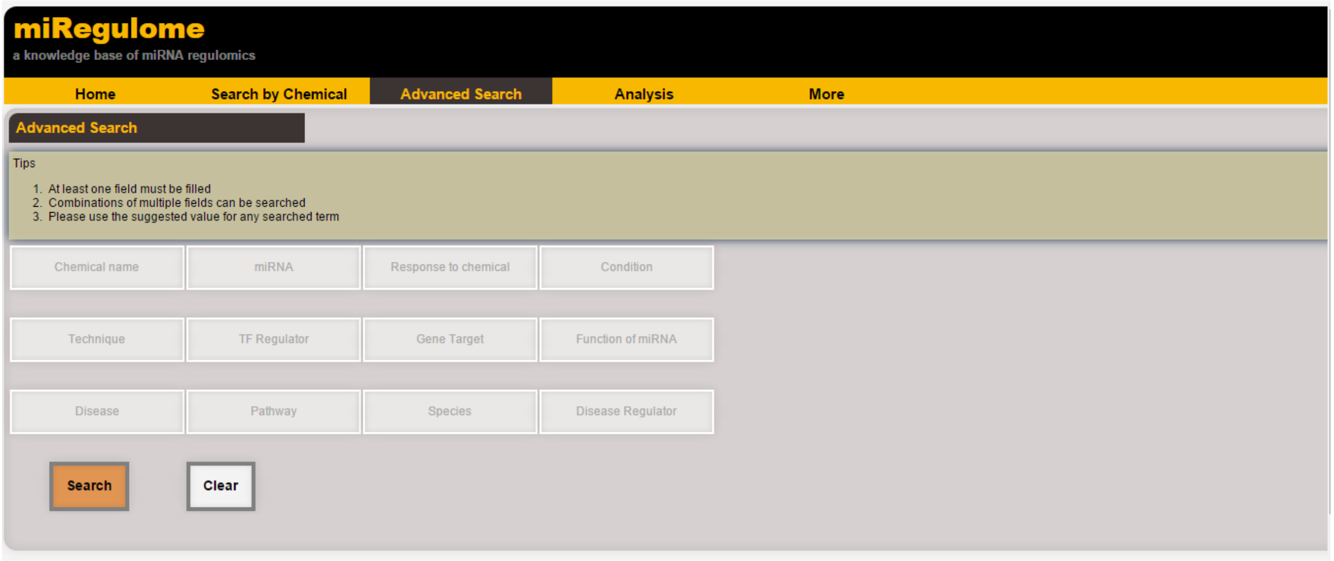

Supplementary Figure S6: The advanced search option of *miRegulome* v1.0. Twelve modules can be searched in combination to obtain specific relationships in a miRNA regulome.

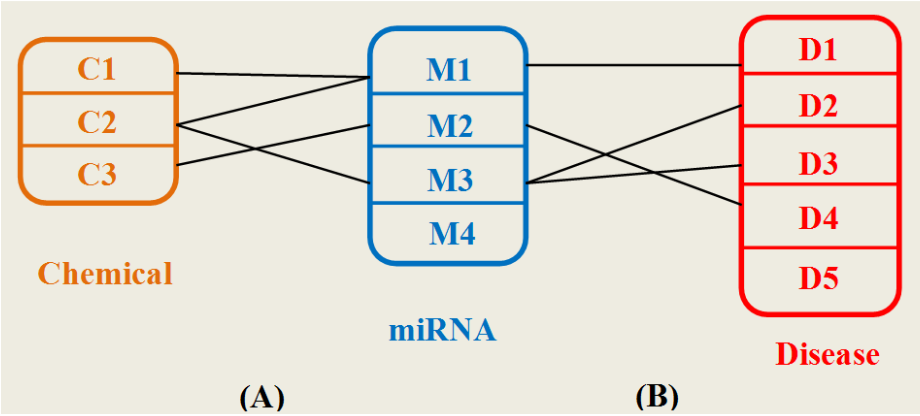

Supplementary Figure S7: Method used in chemical-disease analysis tool. Chemicals are generally upstream regulators in a miRNA. *miRegulome* has curated the PubMed literature citing chemical-miRNA relationships. miRNAs also are significantly up/down regulated in diseases. This analysis tool, allows the user to explore the associations between a chemical to disease via miRNAs. When a user selects a particular chemical, the tool retrieves all the miRNAs associated with the chemical. Thereafter, the tool retrieves all the diseases in which the miRNAs are regulated. In this figure, if a user selects chemical '*C2*' in the *Chemical-Disease* analysis tool, miRNAs *M1* and *M3* are retrieved and subsequently, their associated diseases, *D1*, *D2* and *D3* are retrieved. Finally, the tool shows the top diseases in which, these miRNAs (which are associated with the chemical) have been regulated the most. It does not assert a direct link between the chemical to a disease via the miRNA, rather allows the user to explore and test their hypothesis for indirect associations between the chemical and the disease via the miRNA, by observing the diseases and their subsequent regulations.

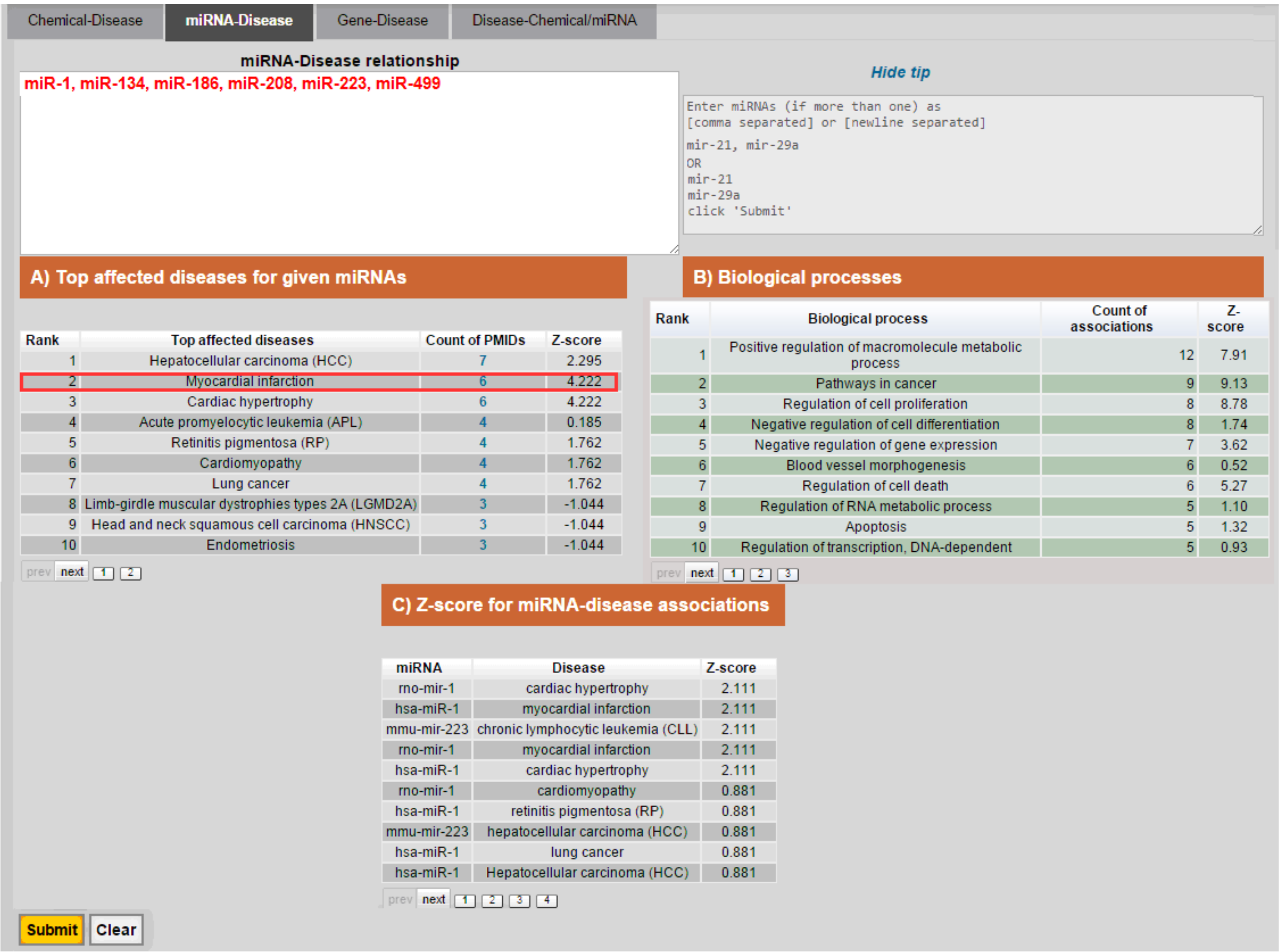

Chemical-Disease | miRNA-Disease | Gene-Disease | Disease-Chemical/miRNA

miRNA-Disease relationship

miR-1, miR-134, miR-186, miR-208, miR-223, miR-499

Hide tip

Enter miRNAs (if more than one) as
[comma separated] or [newline separated]
mir-21, mir-29a
OR
mir-21
mir-29a
click 'Submit'

A) Top affected diseases for given miRNAs

Rank	Top affected diseases	Count of PMIDs	Z-score
1	Hepatocellular carcinoma (HCC)	7	2.295
2	Myocardial infarction	6	4.222
3	Cardiac hypertrophy	6	4.222
4	Acute promyelocytic leukemia (APL)	4	0.185
5	Retinitis pigmentosa (RP)	4	1.762
6	Cardiomyopathy	4	1.762
7	Lung cancer	4	1.762
8	Limb-girdle muscular dystrophies types 2A (LGMD2A)	3	-1.044
9	Head and neck squamous cell carcinoma (HNSCC)	3	-1.044
10	Endometriosis	3	-1.044

prev next 1 2

B) Biological processes

Rank	Biological process	Count of associations	Z-score
1	Positive regulation of macromolecule metabolic process	12	7.91
2	Pathways in cancer	9	9.13
3	Regulation of cell proliferation	8	8.78
4	Negative regulation of cell differentiation	8	1.74
5	Negative regulation of gene expression	7	3.62
6	Blood vessel morphogenesis	6	0.52
7	Regulation of cell death	6	5.27
8	Regulation of RNA metabolic process	5	1.10
9	Apoptosis	5	1.32
10	Regulation of transcription, DNA-dependent	5	0.93

prev next 1 2 3

C) Z-score for miRNA-disease associations

miRNA	Disease	Z-score
rno-mir-1	cardiac hypertrophy	2.111
hsa-miR-1	myocardial infarction	2.111
mmu-mir-223	chronic lymphocytic leukemia (CLL)	2.111
rno-mir-1	myocardial infarction	2.111
hsa-miR-1	cardiac hypertrophy	2.111
rno-mir-1	cardiomyopathy	0.881
hsa-miR-1	retinitis pigmentosa (RP)	0.881
mmu-mir-223	hepatocellular carcinoma (HCC)	0.881
hsa-miR-1	lung cancer	0.881
hsa-miR-1	Hepatocellular carcinoma (HCC)	0.881

prev next 1 2 3 4

Submit | Clear

Supplementary Figure S8: The miRNA-Disease association tool in *miRegulome* v1.0. Human miR-1, miR-134, miR-186, miR-208, miR-223, and miR-499 are deregulated in Acute myocardial infarction (PMID: 23641832). When this set of miRNA is used for analysis, the tool ranks Myocardial infarction at rank-2 with count of PMIDs- 6, and Z-score 4.222. The BPs ranked by the tool also correlate with the diseases.

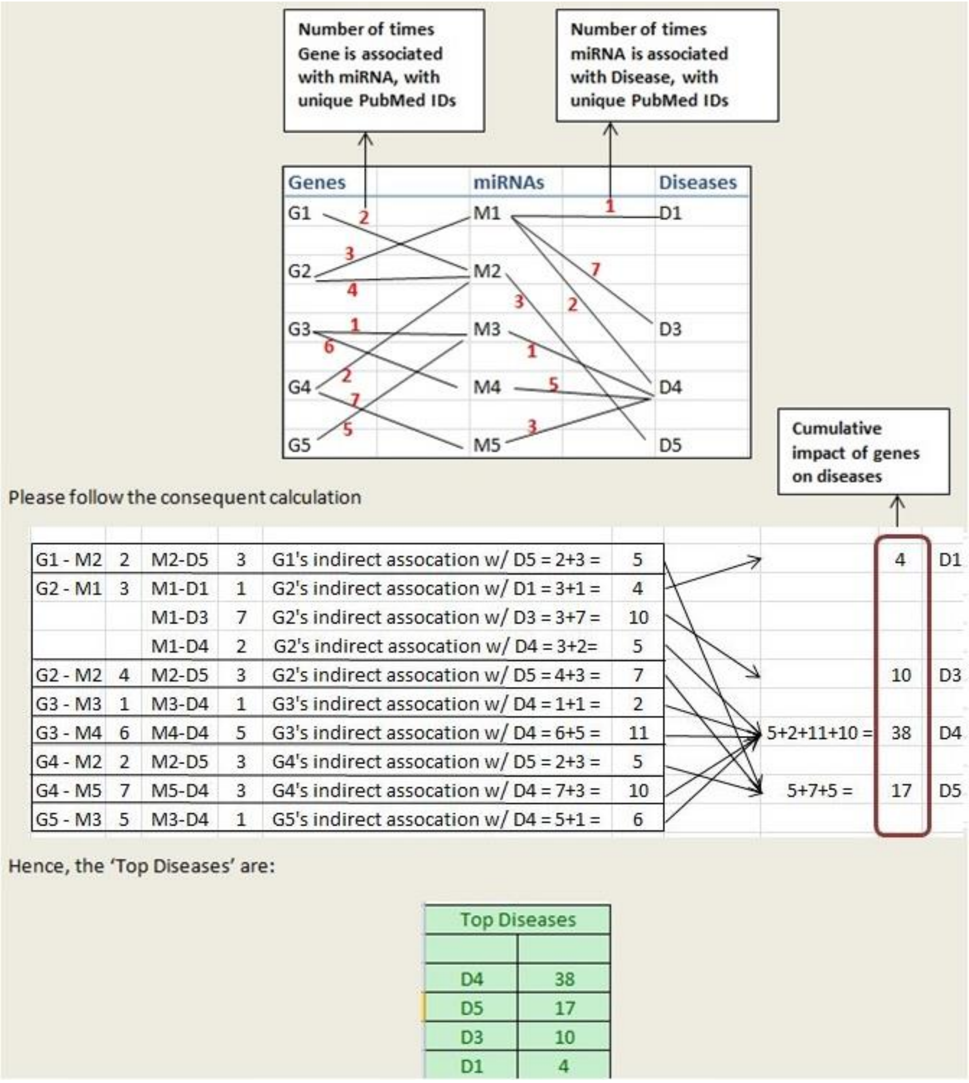

Supplementary Figure S9: Method used in Gene-disease analysis tool. When a list of downstream target genes is entered by user in the input field, the tool searches for miRNAs associated, within the set of genes and counts the number of gene-miRNA associations (i.e. PubMed IDs) recorded in the database. Thereafter, the tool searches and counts the existing relationships (i.e. PubMed IDs) between the observed miRNAs and diseases in which they are regulated, in the database. Following which, the tool ranks the diseases based on their count of PubMed entries, as explained in the Figure. The input set of target genes is G1-G5. The tool pulls up the network of associations between genes (G1-G5) --> miRNAs (M1-M5) --> diseases (D1, D3, D4, D5), where the edge denotes association and regulation and the edge weight denotes the number of unique PubMed IDs supporting it. Thereafter, the tool calculates each gene's indirect association to diseases via the miRNA by counting the unique PubMed IDs, as seen in the tabular calculation in the Figure. Consider Gene G3, which is a target gene for miRNA M3 and M4. The number of PubMed IDs supporting these associations is 1 and 6 respectively. Thereafter, the tool searches for diseases in which these miRNAs M3 and M4 are regulated. The disease is D4, with the number of unique PubMed IDs supporting it, being 1 and 5 by M3 and M4, respectively. This calculation is done for all genes. Finally, based on the cumulative counts contributing to the association/regulation between miRNA-Disease, a sorted list of diseases is generated. The list of diseases suggest that, given the list of input target genes and the corresponding miRNAs involved therein, it is plausible that these diseases would have highest regulation of the

miRNAs and genes in them. In this Figure, disease D4, has the highest count of PubMed IDs contributing to the association/regulation of given target genes, and the miRNAs involved therein, based on the generated network. The tool does not assert a relationship between the entered target genes and diseases. It is left to the user to investigate it further, using other tools, such as Analysis tool, when species of miRNA, tissue, gene name and disease name can be entered to decipher any relationships. The tool merely highlights the top diseases indirectly associated with the genes entered, via the miRNAs.

Supplementary Table S1: Evaluation of miRNA-Disease tool of *miRegulome* v1.0 using ten sets of miRNAs for various diseases taken from ten different PubMed articles that are not incorporated for this version of *miRegulome*. Out of ten tested miRNA sets, in 8 cases (not highlighted), the PubMed mentioned diseases corresponding to the miRNA sets are ranked within the top 10.

PMIDs	Set of human miRNAs	Disease association in PMID	*miRegulome* tool	*miRegulome* predictions	*miRegulome* ranks	*miRegulome* Association scores	Z-scores
23196705	miR-18a, miR-19a, miR-20a, miR-30a, miR-103b, miR-126, miR-192, miR-1287	Breast cancer	miRNA-Disease	Breast cancer	**4**	4	-0.511
22213236	miR-21, miR-31, mir-122, miR-221, miR-222, miR-145, miR-146a, miR-200c, miR-223	Hepatocellular carcinoma	miRNA-Disease	Hepatocellular carcinoma	**3**	50	37.821
23391324	let-7d, miR-150, miR-339, miR-342, let-7b, miR-523	Acute myeloid leukemia	miRNA-Disease	Acute lymphoblastic leukemia	14	2	-0.696
23272653	miR-182, miR-200a, miR-200b, miR-200c	Serous epithelial ovarian cancer	miRNA-Disease	Ovarian cancer	**2**	16	7.050
23178446	miR-122, miR-141, miR-155, miR-184, miR-200c, miR-210, miR-224,miR-514	Clear cell renal cell carcinomas	miRNA-Disease	Kidney cancer	10	8	-2.784
23427895	miR-146b, miR-146, miR-221, miR-222, miR-375, miR-551b, miR-181, miR-99b	Papillary thyroid carcinoma	miRNA-Disease	Papillary thyroid carcinoma	**3**	12	5.288
23207443	miR-21, miR-451, miR-486, miR-205, miR-26b	Lung Cancer	miRNA-Disease	Lung Cancer	**5**	20	18.287
22768114	miR-34a, miR-34b, miR-34c, miR-146b, miR-208a, miR-221, miR-381, miR-125b, miR-193a, miR-193b, miR-378a	Myotonic Dystrophy Type-2	miRNA-Disease	Duchenne muscular dystrophy	11	9	-3.132
23617747	miR-9, miR-200c	Metastatic Breast cancer	miRNA-Disease	Breast cancer	**1**	8	6.682
23641832	miR-1, miR-134, miR-186, miR-208, miR-223, miR-499	Acute myocardial infarction	miRNA-Disease	Myocardial infarction	**2**	6	4.222

Supplementary Table S2: Comparison of miR-Disease tool of *miRegulome* v1.0 and TAM (http://210.73.221.6/tam). All top 15 diseases ranked by miR-Disease tool are presented for a specific set of miRNA that is associated with the specific disease as mentioned in the corresponding PubMed literature. In TAM analysis, same sets of miRNAs were used and the diseases ranked by TAM along with the p-values are given. The diseases that are associated with the specific miRNA sets (as per the PMID) and matched by both the tools are highlighted. Diseases that are ranked within first 10 by the miRegulome tool are highlighted with green and that are above this cut-off are highlighted with red.

		***miRegulome* (miR-Disease tool) Results and Ranks**			**TAM Results**		
**PubMed/Disease**	**Input miRNAs from the PMID**	**Diseases**	**Ranks**	**Z-scores**	**Diseases**	**Ranks**	**p-values**
**23196705 Breast cancer**	miR-18a miR-19a miR-20a miR-30a miR-103b miR-126 miR-192 miR-1287	Lung cancer	1	10.392	Lung neoplasms	5	2.57E-05
		Colorectal cancer	2	12.322	Colorectal neoplasms	37	0.0491
		Hepatocellular carcinoma	3	8.280	Carcinoma,Hepatocellular	26	4.57E-03
		**Breast cancer**	**4**	**-0.511**	**Breast neoplasms**	**32**	**0.0168**
		Medulloblastoma	5	-0.511	Medulloblastoma	2	1.25E-05
		Prostate cancer	6	-1.740	Prostatic neoplasms	62	0.5016
		Glioma	7	-1.044	Glioma	36	0.0275
		Anaplastic thyroid carcinoma	8	-1.044	NA		
		Kidney cancer	9	-1.044	NA		
		Endometriosis	10	-1.044	Endometriosis	33	0.0215
		Acute myeloid leukemia	11	-1.044	Leukemia,Myeloid,Acute	15	3.14E-04
		Malignant lymphoma	12	-1.044	Lymphoma	17	4.04E-04
		Ulcerative colitis	13	-0.696	NA		
		Pancreatic ductal adenocarcinoma	14	-0.696	Pancreatic neoplasms	60	0.4629
		Chronic myeloid leukemia	15	-0.696	NA		
**22213236 Hepatocellular carcinoma**	miR-21 miR-31 mir-122 miR-221 miR-222 miR-145 miR-146a miR-200c miR-223	Colorectal cancer	1	58.552	Colorectal neoplasms	29	1.32E-03
		Breast cancer	2	37.818	Breast neoplasms	9	1.65E-05
		**Hepatocellular carcinoma**	**3**	**37.821**	**Carcinoma, Hepatocellular**	**2**	**9.70E-07**
		Pancreatic cancer	4	32.183	Pancreatic Neoplasms	3	1.83E-06
		Prostate cancer	5	20.947	Prostatic neoplasms	5	3.97E-06
		Lung cancer	6	16.732	Lung neplasms	45	1.06E-02
		Glioblastoma	7	13.547	Glioblastoma	30	1.32E-03
		Bladder cancer	8	4.427	Urinary Bladder Neoplasms	80	0.1046
		Gastric cancer	9	6.355	Stomach neplasms	4	2.73E-06
		Cardiac hypertrophy	10	3.549	Cardiomaypathy, Hypertrophy	75	0.0651
		Oral Squamous Cell Carcinoma (OSCC)	11	-1.185	Carcinoma,Oral	28	1.32E-03
		Papillary thyroid carcinoma (PTC)	12	6.702	Papillary thyroid carcinoma	18	2.46E-04
		Non-small cell lung cancer	13	6.169	Carcinoma,Non-small cell lung	90	0.2383
		Glioma	14	4.941	Glioma	98	0.3489
		Chronic lymphocytic	15	-1.021	NA		

		leukemia					
**23641832 Acute myocardial infarction**	miR-1<br>miR-134<br>miR-186<br>miR-208<br>miR-223<br>miR-499	Hepatocellular carcinoma	1	2.295	Carcinoma,Hepatocellular	38	0.205
		**Myocardial infarction**	**2**	**4.222**	**Myocardial infarction**	**1**	**3.30E-04**
		Cardiac hypertrophy	3	4.222	Cardiomaypathy,Hypertrophic	3	8.80E-03
		Acute promyelocytic leukemia	4	0.185	NA		
		Retinitis pigmentosa (RP)	5	1.762	Retinal Degeneration	24	0.0859
		Cardiomyopathy	6	1.762	Cardiomyopathy,Dilated	11	0.0221
		Lung cancer	7	1.762	Lung Neoplasams	4	0.0101
		Limb-girdle muscular dystrophies types 2A	8	-1.044	NA		
		Head and neck squamous cell carcinoma (HNSCC)	9	-1.044	Head and neck neoplsams	17	0.0388
		Endometriosis	10	-1.044	Endometriosis	16	9.03E-03
		Chronic lymphocytic leukemia	11	2.111	Leukemia,Lymphocytic, Chronic B-cell	41	0.3318
		Colorectal cancer	12	-1.044	NA		
		Nemaline myopathy (NM)	13	-1.044	Distal Myopathis	14	0.033
		Lupus nephritis	14	-1.044	NA		
		Myeloproliferative disorder	15	-0.696	NA		
**23272653 Serous epithelial ovarian cancer**	miR-182<br>miR-200b<br>miR-200a<br>miR-200c	Breast cancer	1	7.585	NA		
		**Ovarian cancer**	**2**	**7.050**	**Ovarian Neoplasms**	**18**	**1.00E-02**
		Hepatocellular carcinoma	3	3.015	Carcinoma,Hepatocellular	17	1.00E-02
		Lung cancer	4	0.207	Lung Neoplasams	41	0.5392
		Epithelial ovarian cancer	5	-3.828	Ovarian Neoplasms	18	1.00E-02
		Malignant melanoma	6	-3.828	Melanoma	12	1.35E-03
		Cancer	7	0.022	Carcinoma	4	3.17E-05
		Kidney cancer	8	-2.784	Carcinoma,Renal cell	19	0.0126
		Oral Squamous Cell Carcinoma (OSCC)	9	1.947	NA		
		Non-alcoholic fatty liver disease	10	-2.088	Fatty liver	20	0.0221
		Endometriosis	11	-2.088	Endometriosis	16	9.03E-03
		Serous ovarian cancer	12	-2.088	Ovarian Neoplasms	18	1.00E-02
		Colorectal cancer	13	-1.740	NA		
		Psoriasis	15	-1.044	NA		
**23391324 Acute myeloid leukemia**	miR-339<br>let-7d<br>miR-150<br>miR-342<br>let-7b<br>miR-523	Lung cancer	2	-0.163	Lung Carcinoma	22	0.1439
		Cardiac Hypertrophy	1	1.415	NA		
		Colorectal cancer	3	-1.392	Colorectal Neoplasms	11	0.2292
		Hepatocellular carcinoma	4	-1.044	Hepatocellular carcinoma	6	0.099
		Neurodegeneration	5	-1.044	Neurodegenerative Disease	31	0.0509
		Multiple myeloma (MM)	6	-1.044	Multiple myeloma	27	0.0721
		Primary biliary cirrhosis	7	-1.044	NA		
		Limb-girdle muscular dystrophies types 2A	8	-0.696	NA		

		Non-small cell lung cancer	9	-0.696	Lung Carcinoma	22	0.1439
		Uveal melanoma	10	-0.696	Melanoma	26	0.0245
		Nemaline myopathy	11	-0.696	NA		
		Cervical cancer	12	-0.696	NA		
		Serous ovarian cancer	13	-0.696	Ovarian Neoplasms	32	0.099
		**Acute lymphoblastic leukemia**	**14**	**-0.696**	**Leukemia, Myeloid, Acute**	**21**	**0.2593**
		Papillary thyroid carcinoma	15	-0.696	NA		
**23178446 Clear cell renal cell carcinomas**	miR-514 miR-122 miR-141 miR-155 miR-184 miR-200c miR-210 miR-224	Breast cancer	1	13.549	Breast neoplasms	48	0.3483
		Hepatocellular carcinoma	2	11.438	Carcinoma Hepatocellular	5	4.57E-03
		Lung cancer	3	5.821	Lung Neoplasams	45	0.2846
		Diffuse large B-cell lymphoma	4	7.564	Lymphoma,Large B-cell ,Diffuse	13	0.033
		Pancreatic ductal adenocarcinoma	5	2.829	Pancreatic Neoplasms	8	0.0144
		Prostate cancer	6	1.599	Prostatic Neoplasms	52	0.5016
		Pancreatic cancer	7	6.334	Pancreatic Neoplasms	8	0.0144
		Cancer	8	3.524	Carcinoma	6	7.40E-03
		Malignant melanoma	9	-2.784	Melanoma	27	0.0906
		**Kidney cancer**	**10**	**-2.784**	**Carcinoma Renal cell**	**1**	**5.94E-05**
		Epithelial ovarian cancer	11	-2.436	Ovarian Neoplasams	15	0.0403
		Colorectal cancer	12	-2.088	NA		
		Oral Squamous Cell Carcinoma	13	-0.511	Carcinoma,Oral	35	0.1559
		Head and neck squamous cell carcinoma	14	-1.740	NA		
		Ovarian cancer	15	-1.740	Ovarian Neoplasams	15	0.0403
**23427895 Papillary thyroid carcinoma**	miR-99b miR-146b miR-146 miR-221 miR-222 miR-375 miR-551b miR-181	Prostate cancer	1	8.977	Prostatic Neoplasms	4	0.0117
		Pancreatic cancer	2	8.096	Pancreatic Neoplasms	19	0.01865
		**Papillary thyroid carcinoma**	**3**	**5.288**	**Thyroid Neoplasms**	**8**	**0.0547**
		Breast cancer	4	2.828	Breast Neoplasms	24	0.3627
		Hepatocellular carcinoma	5	5.986	Carcinoma Hepatocellular	6	0.02
		Pancreatic ductal adenocarcinoma	6	1.947	Pancreatic Neoplasms	19	0.01865
		Glioblastoma	7	3.524	Glioma	11	0.0904
		Coronary artery disease	8	3.524	NA		
		Facioscapulohumeral muscular dystrophy	9	-2.436	NA		
		Dermatomyositis	10	-2.436	NA		
		Nemaline myopathy	11	-2.436	NA		
		Miyoshi myopathy (MM)	12	-2.436	Mascular Disorders Atrophic	1	7.72E-03
		Limb-girdle muscular dystrophies types 2A	13	-2.436	NA		
		Polymyositis	14	-2.436	NA		
		Glioma	15	-2.088	Glioma	11	0.0904
	miR-26b	Colorectal cancer	1	32.166	Colorectal neoplasms	26	0.034

**23207443 Lung cancer**	miR-21<br>miR-451<br>miR-486<br>miR-205	Hepatocellular carcinoma	2	18.821	Carcinoma Hepatocellular	75	0.5373
		Pancreatic cancer	3	20.748	Pancreatic Neoplasms	48	0.0777
		Breast cancer	4	19.517	Breast Neoplasms	9	6.65E-03
		**Lung cancer**	**5**	**18.287**	**Lung Neoplasms**	**35**	**0.0401**
		Prostate cancer	6	10.556	Prostatic Neoplasms	13	0.0105
		Glioblastoma	7	9.675	Glioblastoma	6	2.09E-03
		Cardiac hypertrophy	8	5.289	Cardiomaypathy,Hypertrophy	29	0.0366
		Bladder cancer	9	4.058	Urinary Bladder Neoplasms	27	0.034
		Esophageal cancer	10	5.985	Esophageal Neoplasms	49	0.0807
		Non-small cell lung cancer	11	5.984	Carcinoma,Non-small cell Lung	3	2.06E-04
		Gastric cancer	12	6.333	Stomach Neoplasms	73	0.4222
		Head and neck squamous cell carcinoma	13	4.754	Head and neck carcinoma	23	0.0268
		Glioma	14	6.333	Glioma	22	0.0188
		Oral Squamous Cell Carcinoma	15	1.947	Oral Carcinoma	49	0.1316
**23617747 Breast cancer**	miR-9<br>miR-200c	**Breast cancer**	**1**	**6.682**	**Breast Neoplasms**	**11**	**0.0403**
		Cancer	2	1.762	Carcinoma	20	0.1494
		Lung cancer	3	-0.696	Lung Neoplasms	28	0.3206
		Oral Squamous Cell Carcinoma	4	-0.696	NA		
		Kidney cancer	5	-0.696	Carcinoma, Renal cell	16	0.0939
		Non-alcoholic fatty liver disease	6	-0.696	NA		
		Epithelial ovarian cancer	7	-0.696	Ovarian Neoplasams	3	0.02
		Melanoma	8	-0.696	Melanoma	10	0.0374
		Colorectal cancer	9	-0.696	NA		
		Malignant melanoma	10	-0.696	Melanoma	10	0.0374
		Testicular germ cell tumor	11	-0.696	Prostatic Neoplasms	26	0.2064
		Lupus nephritis	12	-0.696	NA		
		Ovarian cancer	13	-0.696	Ovarian Neoplasams	3	0.02
		Serous ovarian cancer	14	-0.696	Ovarian Neoplasams	3	0.02
		Endometrial cancer	15	-0.696	Endometrial Neoplasms	8	0.033
**22768114 Myotonic dystrophy type-2**	miR-378a<br>miR-34a<br>miR-34b<br>miR-34c<br>miR-146b<br>miR-208a<br>miR-221<br>miR-381<br>miR-125b<br>miR-193a<br>miR-193b	Pancreatic cancer	1	8.281	Pancreatic Neoplasms	8	1.30E-03
		Malignant melanoma	2	1.088	Melanoma	14	5.02E-03
		Colorectal cancer	3	2.133	Colorectal neoplasms	4	1.47E-04
		Neuroblastoma	4	5.637	Neuroblastoma	61	0.2175
		Hepatocellular carcinoma	5	5.637	Carcinoma Hepatocellular	34	0.0406
		Prostate cancer	6	5.638	Prostatic Neoplasms	6	1.96E-04
		Non-small cell lung cancer	7	-0.326	Carcinoma Non-small cell lung	66	0.2612
		Melanoma	8	0.022	Melanoma	14	5.02E-03
		Breast cancer	9	0.022	NA		
		Papillary thyroid carcinoma	10	0.022	Papillary thyroid carcinoma	32	0.0367

		**Duchenne muscular dystrophy**	**11**	**-3.132**	**Muscular Disorders, Atrophic**	**15**	**7.72E-03**
		Ovarian cancer	12	-1.207	NA		
		Chronic lymphocytic leukemia	13	1.947	Leukemia,Lymphocytic,Chronic,B-cell	5	1.70E-04
		Oral Squamous Cell Carcinoma	14	-2.784	Carcinoma,Oral	10	1.85E-03
		Glioma	15	-2.436	Glioblastoma	73	0.4804

Supplementary Table S3: Evaluation of Gene-Disease tool of *miRegulome* v1.0 using ten sets of genes for various diseases taken from ten different PubMed articles that are not curated for this version of *miRegulome*.

**PMIDs**	**Set of Genes**	**Disease association in PMID**	***miRegulome* tool**	***miRegulome* predictions**	***miRegulome* ranks**	***miRegulome* Association scores**
19951989	RSF1, DYRK2, YY1, C19orf12, THEM2, TRIO, MYADM, BAIAP2, ROGDI, DNAJB14, BRE, TMEM41A, C9orf64, FAM110A, PCNXL2, REST, C19orf62, C13orf27, ASCC3, SLC1A5, PTPLAD1, MRE11A, GTPBP10, BX118737, SERPINI2, CREB1, CCDC53, USP48, ZSCAN2	Non-Small-Cell Lung Cancer	Gene-Disease	Lung cancer	5	215
21610147	APC, CDH1, MGMT, DCC, RASSF1A, AIM1	Lung cancer	Gene-Disease	Lung cancer	5	142
23533275	ACTR3B, APOC1, ATP8A1, C10orf116, Cdk1, CDON, DEGS1, SYMN, DPP4, F12, FEV, GATA3, GSTM3, HIST1H3H, HOXC4, TMEM132A, IGSF1, IGSF6, INHBA, KRT15, LDHB, KIF18B, NCAPG2, MAOA, MT1F, OIP5, PPP3CB, QPRT, TPX2	Prostate cancer	Gene-Disease	Prostate cancer	8	62
23536436	CD36, DHRS13, DUSP2, FAM198B, FKBP5, GLT25D2, GZMB, IL1B, ITGAM, ITPRIPL2, MYBL1, NEAT1, NUDT16, P2RY10, PDE4D, PDZK1IP1, SH2D2A, VSIG10	Colorectal cancer	Gene-Disease	Colorectal cancer	1	292
23599765	MYF6, SIX6, SOX1, RARB, BCL2, PHOX2A, FOLX2	Non-small cell lung cancer	Gene-Disease	Lung cancer	7	256
23606240	COL2A1, ATP4B	Gastric cancer	Gene-Disease	Colorectal cancer	1	99
23591077	CLDN1, CLDN10, MMP2, c-fos	Lung cancer	Gene-Disease	lung cancer	6	39
23651824	XPO1, GABPB2, RANBP17, KALRN, XPO5	Heart failure	Gene-Disease	Cardiac hypertrophy	10	27
23648477	FGF18, BCL2, PRC1, MMP9, SERF1a	Breast cancer	Gene-Disease	Breast cancer	4	380
21733826	TP53, CTAG1B, CAGE, ANNX, SOX2	Lung cancer	Gene-Disease	Lung cancer	6	387
